# Prevalence and correlates of roll-your-own cigarette smoking among South African adults during 2010–2011 and 2017–2018

**DOI:** 10.18332/tid/154798

**Published:** 2022-11-03

**Authors:** Lungile Nkosi, Israel T. Agaku, Olalekan Ayo-Yusuf

**Affiliations:** 1 The Africa Centre for Tobacco Industry Monitoring and Policy Research, Sefako Makgatho Health Sciences University, Ga-Rankuwa, South Africa; 2 Department of Oral Health Policy and Epidemiology, Harvard School of Dental Medicine, Boston, United States; 3 School of Health Systems and Public Health, University of Pretoria, Pretoria, South Africa

**Keywords:** South Africa, tobacco price, manufactured cigarettes, smoking, roll-your-own

## Abstract

**INTRODUCTION:**

The increasing use of roll-your own (RYO) cigarettes has been documented globally, but there are no recent data from South Africa, particularly among youths and low-income groups. We assessed changes in prevalence and correlates of RYO smoking among South African adults during 2010–2011 and 2017–2018, and explored expenditure differences between daily smokers of RYO and manufactured cigarettes.

**METHODS:**

Nationally representative data of South Africans aged ≥16 years used in this study were from the South African Social Attitudes Survey (SASAS) for 2010–2011 (n=6116), and 2017–2018 (n=5799). Current RYO cigarette use included daily and non-daily use. Annual expenditures were estimated based on typical usage patterns for daily users. Descriptive and multivariable analyses were performed using Stata Version 15 with the level of statistical significance set at p<0.05.

**RESULTS:**

The prevalence of ever RYO cigarette use increased from 6.5% (95% CI: 5.6–7.5) during 2010–2011, to 8.5% (95% CI: 7.0–10.0) during 2017–2018 (p=0.026). Current RYO cigarette use prevalence however remained largely unchanged when we compared 2010–2011 to 2017–2018 (5.2% vs 6.3%, p=0.544). During 2017–2018, current RYO cigarette use prevalence was highest among men (11.6%), those who self-identified as Coloreds (11.1%), people aged 25–34 years (7.8%), those with no schooling (7.5%), and those unemployed (9.8%). Annual expenditures associated with typical patterns of daily RYO cigarette smoking were substantially less than for smoking of manufactured cigarettes.

**CONCLUSIONS:**

The prevalence of ever RYO cigarette use increased between 2010–2011 and 2017–2018. Current RYO cigarette use during 2017–2018 was more prevalent among Coloreds, Black Africans, youths, those with lower education, and the unemployed. This study’s findings highlight the need to harmonize taxation of cigarettes and RYO cigarettes, and to intensify implementation of evidence-based tobacco control and prevention interventions in South Africa.

## INTRODUCTION

The use of roll-your-own (RYO) cigarettes has been increasing in several developed, and developing countries, even as smoking of manufactured cigarettes has declined^1-5^. In South Africa (SA), Ayo-Yusuf and Olutola^6^ found that the use of RYO cigarettes among current smokers had increased from 20% to 28.8% between 2007 and 2010. Although RYO cigarettes are perceived as a safer alternative to manufactured cigarettes, their combustion yields the same harmful toxins and carcinogens that have been shown to cause damage to every organ in the body^7^.

RYO smoking may worsen socioeconomic disparities in tobacco-related morbidity and mortality because of its higher prevalence among groups with lower socioeconomic status, including Black African males from rural areas and individuals with lower education. The most common, and strongest contributing factor to RYO cigarette smoking across several studies was its relatively lower costs compared to manufactured cigarettes^1-4,6,8,9^. Other factors associated with RYO smoking include perceived taste/satisfaction, and perceptions of lower health risks^10^. The increasing availability of flavored RYO tobacco in recent years has potential to increase the prevalence of RYO use among youths because of the increased ease of use and product appeal^4,10-12^.

No nationally representative study has been published in the past 10 years on the sociodemographic profile of RYO cigarette users within the South African context. In the light of the evolving regulatory landscape, there is a need for up-to-date data on current use patterns, as well as examination of whether shifts have occurred in the key user segments. Furthermore, while it is well acknowledged anecdotally that RYO cigarettes are cheaper than cigarettes in South Africa, the potential for smoker population down-trading from cigarettes to RYO cigarette smoking has not been evaluated empirically within the South African context, especially in the context of increasing availability of cheaper and/or illicitly traded cigarettes^13^ and increasing unemployment in South Africa that could make cigarettes generally unaffordable^14,15^. This study therefore sought to answer three key questions: 1) ‘How did the percentage and composition of South African adults reporting ever and current use of RYO cigarettes change between the periods 2010–2011 compared to 2017–2018, and what factors were associated with current RYO cigarette use in the more recent period?’; 2) ‘How did the demographic profile of those smoking RYO cigarettes exclusively compare to those smoking manufactured cigarettes exclusively, as well as those smoking both products?’; and 3) ‘What were expenditure differences between daily smokers of RYO cigarettes vs manufactured cigarettes in South Africa during 2018?’.

## METHODS

### Data sources

Data were from four waves of the South African Social Attitudes Survey (SASAS): 2010 (n=3112), 2011 (n=3004), 2017 (n=3063), and 2018 (n=2736). To increase sample size for subgroup analysis, we combined data for the 2010 and 2011 waves (henceforth, 2010–2011 cycle, pooled n=6116), as well as the 2017 and 2018 waves (henceforth, 2017–2018 cycle, pooled n=5799). SASAS is a nationally representative cross-sectional, face-to-face, household survey of the South African civilian adult population aged ≥16 years, which is conducted by the Human Sciences Research Council. People living in mental institutions, prisons, nursing homes, military barracks, and dormitories are excluded. The survey employs a multi-stage probability sampling method^16^. Each SASAS round of interviewing consists of a sub-sample of 500 enumeration areas (i.e. primary sampling unit). To ensure the sample was diverse and representative, stratification of enumeration areas was done by province, residence (rural vs urban) and race.

Inputs used to estimate annual expenditures associated with cigarette smoking came from SASAS (average cigarettes smoked per day) and other sources described below. Based on previous reports indicating a quite simple, price-minimizing process of RYO cigarette smoking among most users in South Africa where ‘pipe tobacco is wrapped in newspaper and used as roll-your-own cigarette’^6,17^, we estimated only the associated costs of pipe or RYO tobacco (and not the additional costs of the rolling paper). We included prices for not only products labelled as actual RYO tobacco, but also pipe tobacco being marketed for use as hand-rolled cigarettes. Including both types of products ensured we adequately captured the diversity of products of different costs, sources, and user profiles. To derive cost estimates for pipe or RYO tobacco, we accessed South Africa Yellow Pages listing of tobacco shops in South Africa^18^. From those selling pipe or RYO tobacco, we extracted information on name of vendor, name of pipe/RYO product, description, size of packet, and the price. The most recurring pouch size was 50 g of pipe or RYO tobacco, the median price of which was ZAR 96.8 (1000 South African Rand about US$55, currently).

### SASAS Measures

*Sociodemographic variables*

Sociodemographic variables included age (16–24; 25–34; 35–44; 45–64; and ≥65 years), gender (male or female), race (self-identifying as Black African, Colored, White, or Asian/Indian), education level (no schooling/less than primary school, completed primary school, some high school, completed high school, tertiary education or equivalent), and employment status (employed, unemployed, not in the workforce).

*Tobacco product use behavior*

Ever and current use of the following products was assessed: manufactured cigarettes, RYO cigarettes, hubbly-bubbly/hookah/waterpipe, and e-cigarettes. Ever users were defined as persons who indicated they used the particular tobacco product ‘currently every day’, ‘currently some days’, ‘stopped completely, less than 6 months’, ‘stopped completely, more than 6 months’. Current users were those who answered ‘currently every day’ or ‘currently some days’.

Number of cigarettes smoked per day (CPD) was assessed with the question ‘On the days that you smoke, on average, how many manufactured cigarettes, (excluding hand rolled cigarettes) do you smoke per day?’. Participants could type in a numeric response. We used this question to estimate the median number of cigarettes smoked per day among users of manufactured cigarettes as well as among users of RYO cigarettes (77.9% of RYO smokers also smoked manufactured cigarettes in 2018; we assumed their smoking intensity when using hand-rolled cigarettes was similar to when using manufactured cigarettes).

Associated costs from the last cigarette purchase made was assessed in SASAS with the questions: 1) ‘How much did you pay for your last cigarette purchase, per stick/individual cigarette?’ (For those who bought single sticks); and 2) ‘How much did you pay for your last cigarette purchase, per pack?’ (For those who bought cigarettes in a pack).

### Data analysis

All statistical analyses were performed using Stata Version 15 (Stata Corp, College Station, TX, USA). Data were weighted to yield nationally representative estimates. Descriptive analyses were performed to explore whether the percentage of South African adults reporting ever and current use of RYO cigarettes changed between 2010–2011 and 2017– 2018; whether the composition of current RYO cigarette smokers changed between the two specified periods; and how the demographic profile of those smoking RYO cigarettes exclusively compared with those smoking manufactured cigarettes exclusively, and those smoking both RYO and manufactured cigarettes. Group differences were tested using chi-squared statistic at the 5% alpha level. List-wise deletion was performed for missing records. For the study aim comparing the demographic characteristics of exclusive RYO cigarette smokers, exclusive manufactured cigarette smokers, and dual users of both products, we combined data across all four waves (2010, 2011, 2017, and 2018) to ensure adequate sample size for the exclusive RYO cigarette smoker category. For the demographic profile of exclusive RYO cigarette smokers, the denominator was survey participants who reported that they currently smoked RYO but not manufactured cigarettes; exclusive smokers of manufactured cigarettes were those who reported they currently smoked manufactured cigarettes but not RYO, while dual smokers of RYO and manufactured cigarettes comprised survey participants who reported they currently smoked both RYO and manufactured cigarettes.

Adjusted prevalence ratios (APRs) were calculated in a multivariable Poisson regression model to explore factors associated with current RYO cigarette use using the 2017–2018 pooled data; independent variables assessed were province, age, gender, employment status, residence, race, as well as use of other tobacco products such as manufactured cigarettes, hookah, cigars, e-cigarettes, and snuff. Statistical significance was set at p<0.05 for the final model. Unless otherwise specified in the context of exclusive use between RYO and manufactured cigarettes, all mentions of RYO cigarette use (ever or current) are mutually inclusive of manufactured cigarettes/other tobacco products (i.e. they describe RYO use, regardless of whether the individuals used manufactured cigarettes or any other tobacco product).

*Expenditure difference between daily smokers of RYO versus manufactured cigarettes*

For daily cigarette smokers who bought manufactured cigarettes in sticks, the daily cost of smoking cigarettes was the cost of a single stick multiplied by the number of cigarettes smoked per day. For those who bought in packs, it was cost of a single pack multiplied by (number of cigarettes smoked per day/20). Monthly expenditures were extrapolated by multiplying daily costs by 30, annual expenditures by multiplying monthly expenditures by 12.

RYO cigarette smoking is more subject to variability in the amount of tobacco consumed and the overall expenditure, depending on the size of the paper, how it is rolled (fairly tight vs loose/under filled), as well as whether the smoker used other accessories such as rolling machines, rolling paper, filter tips, filter tubes, lighters, holders for papers or tobacco, aroma cards, or other related accessories. To estimate the annual expenditure associated with daily RYO cigarette smoking, we first determined the expected number of cigarettes to be smoked in a year by multiplying the median CPD determined among RYO smokers (6 sticks, from 2018 SASAS) by 365, yielding 2190 cigarettes. We then calculated the number of 50 g bags of RYO tobacco (i.e. the most recurring size) that would need to be bought in a year to meet demand by dividing 2190 by the minimum number of RYO cigarettes that could be rolled from a 50 g tobacco pouch. Multiplying the number of RYO bags smoked in a year by the cost of 1 bag yielded the total estimated annual expenditure associated with daily smoking of RYO cigarettes.

To account for the potential variability in the minimum number of cigarettes that could be rolled from a 50 g tobacco pouch, we applied varying paper sizes as specified in the British Standard International Organization of Standardization statistics^19^, an internationally recognized benchmark. These were: 1) a 70 mm cigarette paper which consumes 0.75 g of tobacco per cigarette stick, equivalent to a yield of 66 rolled cigarettes per 50 g pouch or 33 sets of 50 g pouches in a year (i.e. 2190/66); 2) a 79 mm cigarette paper which consumes 0.88 g of tobacco per cigarette stick, equivalent to a yield of 56 rolled cigarettes per 50 g pouch, or 39 sets of 50 g pouches in a year; and 3) a 100 mm cigarette paper which consumes 1.18 g of tobacco per cigarette stick, equivalent to a yield of 42 rolled cigarettes per 50 g pouch, or 52 sets of 50 g pouches in a year.

## RESULTS

### Sociodemographic characteristics of the study population

In the 2017–2018 cycle of SASAS, the population comprised mostly Black Africans (78.6%), whereas the smallest racial group was Indian/Asian (2.9%) (Table 1). By age, 50.6% of the population was aged 16–35 years. Gender composition was similar between males (50.0%) and females (50.0%). Over a third of the population (37%) had completed high school, and only 3.7% of participants reported no formal schooling. The significant difference between the two pooled periods of surveys was that more people had completed high school, and more were unemployed in 2017–2018 compared to 2010–2011.

**Table 1:** Characteristics of study participants in SASAS between 2010–2011 and 2017–2018

*Characteristics*	*2010–2011*		*2017–2018*		*p ^a^*
	*n*	*%*	*n*	*%*	
**Total**	6116	100	5799	100	
**Race**					
Black African	3664	76.7	3596	78.6	0.542
Colored	1037	9.4	904	9.1	
Indian/Asian	624	2.9	685	2.9	
White	788	11.0	611	9.5	
**Age** (years)					
16–24	1184	27.2	931	24.1	0.128
25–34	1406	25.6	1202	26.6	
35–44	1224	18.2	1126	19.2	
45–54	1039	13.7	861	13.3	
55–64	659	8.3	823	9.2	
≥65	599	7.1	856	7.8	
**Gender**					
Male	2508	47.6	2321	50.0	0.098
Female	3607	52.4	3478	50.0	
**Education level**					
No school	260	3.8	261	3.7	<0.001
Primary	931	14.2	857	11.8	
Some high school	2161	37.2	1985	36.8	
High school completed	1751	31.6	1900	37.0	
Tertiary	892	13.2	640	10.0	
Other/don’t know	0	0.0	34	0.7	
**Employment status**					
Employed	2090	31.7	1549	26.4	<0.001
Unemployed	1616	22.8	1718	27.8	
Not in the workforce	2234	45.5	2138	45.8	

All percentages (%) are weighted, and all raw counts (n) are unweighted.

a The p-values are from chi-squared tests of independence and are testing whether the sociodemographic composition differed between the two time periods (i.e. 2010–2011 vs 2017–2018).

All tests were two-tailed and deemed significant at p<0.05.

### Prevalence and correlates of RYO cigarette smoking between 2010–2011 and 2017–2018

Ever RYO cigarette smoking in the overall population increased from 6.5% during 2010–2011, to 8.5% during 2017–2018 (p=0.026) (Table 2). During 2017–2018, the prevalence of ever RYO cigarette use was highest among those who self-identified as Coloreds at 13.1%, followed by Black Africans at 8.4%, and lowest among Indians/Asians (5.6%) and Whites (5.8%). By age, ever use prevalence of RYO cigarette smoking was just over 9% in the age groups 16–24 (9.2%), 25–34 (9.4%), and 55–64 years (9.5%), and lowest among those aged ≥65 years (7.0%). Ever use of RYO cigarettes was over five-fold higher among men (14.4%) than women (2.7%). Participants with primary school education had the highest prevalence (12.1%) of ever use of RYO cigarette smoking, whereas those with tertiary education had the lowest prevalence (4%). Unemployed participants had the highest prevalence (12.5%) of ever smoking RYO cigarettes, compared to employed participants at 8.1%.

**Table 2:** Percentage of South African adults aged ≥16 years who reported ever ^a^ and current ^b^ use of roll-yourown cigarettes between 2010–2011 and 2017–2018

*Characteristics*	*Ever use*			*Current use*		
	*2010–2011*	*2017–2018*	*p^c^*	*2010–2011*	*2017–2018*	*p^c^*
	*% (95% CI)*	*% (95% CI)*		*% (95% CI)*	*% (95% CI)*	
**Total**	6.6 (5.6–7.5)	8.5 (7.0–10.0)	0.026	5.2 (4.3–6.0)	6.3 (5.3–7.9)	0.544
**Race**						
Black African	7.0 (5.9–8.2)	8.4 (6.7–10.2)	0.178	5.5 (4.5–6.6)	6.6 (5.1–8.1)	0.243
Colored	9.9 (7.4–12.5)	13.1 (8.7–17.5)	0.209	8.3 (5.8–10.8)	11.1 (7.0–15.2)	0.242
Indian/Asian	2.7 (0.1–5.4)	5.6 (2.2–9.0)	0.210	2.3 (–0.3–5.0)	2.0 (0.9–3.1)	0.828
White	**1.5 (0.4–2.6)**	**5.8 (2.6–9.0)**	**0.001**	**0.5 (0.1–1.0)**	**4.2 (1.3–7.1)**	**0.000**
**Age** (years)						
16–24	6.6 (4.5–8.7)	9.2 (5.9–12.5)	0.175	5.8 (3.8–7.7)	7.0 (4.5–9.4)	0.438
25–34	6.1 (4.3–7.8)	9.4 (6.1–12.7)	0.054	**4.2 (2.9–5.5)**	**7.8 (4.7–11.0)**	**0.015**
35–44	6.7 (4.8–8.7)	7.2 (4.9–9.5)	0.764	5.3 (3.5–7.1)	5.9 (3.6–8.1)	0.685
45–54	6.5 (4.4–8.5)	7.7 (4.7–10.6)	0.499	5.4 (3.4–7.3)	6.8 (4.0–9.7)	0.394
55–64	9.6 (6.2–13.1)	9.5 (6.5–12.6)	0.956	7.4 (4.4–10.4)	6.5 (3.9–9.1)	0.660
≥65	4.6 (2.2–6.9)	7.0 (4.0–10.0)	0.195	2.9 (1.3–4.6)	3.2 (1.1–5.2)	0.859
**Sex**						
Male	11.3 (9.6–13.1)	14.4 (11.6–17.2)	0.062	9.0 (7.4–10.6)	11.6 (9.2–14.1)	0.071
Female	2.3 (1.6–2.9)	2.7 (1.9–3.4)	0.408	1.6 (1.1–2.2)	1.6 (1.1–2.2)	0.977
**Education level**						
No school	10.0 (5.4–14.6)	9.7 (3.3–16.2)	0.942	7.6 (3.7–11.5)	7.5 (1.5–13.5)	0.966
Primary	11.8 (9.0–14.7)	12.1 (8.8–15.3)	0.908	9.9 (7.2–12.7)	8.4 (5.7–11.1)	0.429
Some high school	**5.5 (4.2–6.8)**	**7.8 (6.0–9.5)**	**0.039**	4.8 (3.6–6.1)	6.3 (4.7–7.9)	0.156
High school completed	**5.8 (4.1–7.6)**	**9.3 (6.3–12.3)**	**0.037**	**4.0 (2.5–5.4)**	**7.1 (4.4–9.8)**	**0.025**
Tertiary	4.6 (2.6–6.6)	4.0 (1.5–6.5)	0.720	3.2 (1.5–4.9)	3.7 (1.2–6.2)	0.755
**Employment status**						
Employed	7.5 (5.8–9.2)	8.1 (5.5–10.7)	0.682	5.7 (4.2–7.2)	6.8 (4.2–9.4)	0.454
Unemployed	**8.1 (6.1–10.0)**	**12.5 (9.2–15.9)**	**0.015**	**5.7 (4.2–7.2)**	**9.8 (6.6–13.0)**	**0.011**
Not in the workforce	5.4 (4.0–6.9)	6.0 (3.7–8.2)	0.682	4.7 (3.3–6.0)	4.5 (3.0–6.1)	0.901

All percentages (%) are weighted, and all raw counts (n) are unweighted.

a Ever use was defined as persons who indicated they used the particular tobacco product ‘currently every day’, ‘currently some days’, ‘stopped completely, less than 6 months’, ‘stopped completely, more than 6 months’.

b Current was defined as persons who indicated they used the particular tobacco product ‘currently every day’, or ‘currently some days’.

c The p-values are testing for differences within each row when comparing 2017–2018 vs 2010–2011 for ever or current use of roll-your-own cigarettes, as appropriate.

A p<0.05 was deemed statistically significant.

The prevalence of current RYO cigarette use during 2010–2011 was 5.2% and did not change significantly compared to 2017–2018 (6.3%, p=0.544). During 2017–2018, the prevalence of current RYO cigarette use was also highest among Coloreds at 11.1%, followed by Black Africans at 6.6% but lowest among Indians/Asians at 2.0%. The prevalence of RYO cigarette current use was highest (7.8%) in the age group 25–34 years, followed by the 16–24 years age group (7.0%), but lowest among the oldest age group ≥65 years (3.2%). Current use of RYO cigarettes was over seven-fold higher among men (11.6%) than women (1.6%). Participants with primary school education had the highest prevalence (8.4%) of current use of RYO cigarette smoking, followed by participants with no schooling (7.5%); those with tertiary education had the least prevalence (3.7%). Unemployed participants had the highest prevalence (9.8%) of current RYO cigarette smoking, compared to employed participants at 6.8%.

Within segmentation analyses of RYO cigarette smokers during 2010–2011 versus 2017–2018, results showed shifts in the composition of current RYO cigarette smokers by employment and education, but not by race, gender, and age. Specifically, of current RYO cigarette smokers, the percentage of unemployed individuals doubled between 2010–2011 (24.8%) and 2017–2018 (41.5%); correspondingly, the percentage of employed individuals decreased, as shown in Table 3. Similarly, by education, an increase was seen in the composition of current RYO cigarette smokers who reported having completed high school, increasing from 24.1% in 2010–2011, to 39.6% in 2017–2018.

**Table 3:** Composition of current smokers of roll-your-own cigarettes by selected demographic characteristics between 2010–2011 and 2017–2018

*Characteristics*	*2010–2011 (n=318) %*	*2017–2018 (n=327) %*	*p ^a^*
**Race**			
Black African	82.3	78.0	0.0593
Colored	15.2	15.2	
Indian/Asian	1.3	0.9	
White	1.2	6.0	
**Age** (years)			
16–24	30.3	25.4	0.4436
25–34	20.8	31.2	
35–44	18.6	16.9	
45–54	14.2	13.7	
55–64	11.9	9.1	
≥65	4.1	3.7	
**Gender**			
Male	83.2	87.7	0.174
Female	16.8	12.3	
**Education level**			
No school	5.6	4.2	0.0183
Primary	27.2	14.9	
Some high school	34.9	34.9	
High school completed	24.1	39.6	
Tertiary	8.2	5.5	
Other/don’t know	0.0	1.0	
**Employment status**			
Employed	34.5	27.2	0.038
Unemployed	24.8	41.5	
Not in the workforce^b^	40.7	31.4	

All percentages (%) are weighted, and all raw counts (n) are unweighted.

a The p-values are from chi-squared tests of independence and are testing whether the sociodemographic composition differed between the two time periods (i.e. 2010–2011 vs 2017–2018). All tests were two-tailed and deemed significant at p<0.05.

b Includes those retired, homemakers, or very disabled and unable to work, or attending school.

### Distinct characteristics associated with RYO versus manufactured cigarette smoking

Striking differences were seen among those who smoked RYO cigarettes and how they smoked it, contrasted to manufactured cigarettes. Within 2018 SASAS, daily use was more common for manufactured cigarettes than RYO cigarettes. Of those reporting ever use of manufactured cigarettes during 2018, 65.3% reported they smoked daily, 21.2% smoked some days, 3.1% had quit within the past 6 months, and 10.4% had quit >6 months ago. For RYO cigarettes, however, the corresponding percentages were 46.9% for daily smoking, 38.9% for smoking on some days, 4.2% for quitting within the past 6 months, and 9.9% for quitting >6 months ago.

Significant differences were seen in the racial composition of exclusive smokers of RYO cigarettes, exclusive manufactured cigarettes, and dual users of RYO and manufactured cigarettes. Within pooled analysis across all four survey waves, exclusive RYO cigarette smokers had a higher composition of Black Africans (88.7%) versus exclusive smokers of manufactured cigarettes (55.3%). Exclusive RYO cigarette smokers also had a higher composition of smokers aged 16–24 years (28.6%) than exclusive smokers of manufactured cigarettes (18.6%). Males comprised most users for both RYO and manufactured cigarettes but made up a higher proportion for RYO cigarette (81.7%) than manufactured cigarette (70.9%) smoking. Individuals with no schooling or with only primary school education made up over a third of current RYO cigarette smokers (38.0%) but only 15.3% exclusive smokers of manufactured cigarettes. For dual smokers of RYO and manufactured cigarettes, this educational composition (i.e. primary school education or less) was 21.2%; other results for this group are shown in Table 4.

**Table 4:** Composition of exclusive RYO cigarette smokers, exclusive manufactured cigarette smokers, and dual smokers of RYO and manufactured cigarettes ^a^, by selected demographic characteristics using pooled data for the four survey cycles combined (2010, 2011, 2017, and 2018)^b^

*Characteristics*	*Exclusive RYO cigarettes (n=128) %*	*Exclusive manufactured cigarettes (n=1803) %*	*RYO and manufactured cigarettes (n=515) %*	*p ^c^*
**Race**				
Black African	88.7	55.3	77.6	<0.001
Colored	9.0	19.6	16.7	
Indian/Asian	0.3	4.9	1.2	
White	2.1	20.2	4.5	
**Age** (years)				
16–24	28.6	18.6	27.1	0.116
25–34	31.7	27.3	25.9	
35–44	9.8	21.0	19.5	
45–54	9.0	17.3	15.0	
55–64	14.4	9.4	9.2	
≥65	6.4	6.4	3.2	
**Gender**				
Male	81.7	70.9	87.0	<0.001
Female	18.3	29.2	13.0	
**Education level**				
No school	8.5	2.2	3.8	0.021
Primary	29.5	13.1	17.4	
Some high school	28.4	38.3	36.4	
High school completed	31.3	34.5	34.0	
Tertiary	1.5	11.7	7.8	
Other/don’t know	0.9	0.2	0.5	
**Employment status**				
Employed	16.1	38.0	33.5	0.076
Unemployed	43.3	34.9	32.6	
Not in the workforce^d^	40.6	27.1	33.8	

All percentages (%) are weighted, and all raw counts (n) are unweighted. RYO: roll-your-own.

a The definition of exclusive or dual use considered only manufactured and RYO cigarettes relative to each other and not in relation to other tobacco product type. Hence, exclusive users of either assessed product type could have been concurrently using other tobacco products not specified. Classifying adults as exclusive RYO smokers, exclusive manufactured cigarette smokers, and dual users of both products required that individuals have complete information regarding their status for both RYO and manufactured cigarette smoking. The sum of exclusive RYO users and dual users of RYO and manufactured cigarettes (i.e. 1218 + 515 = 643) is less than the number reporting current RYO use (645) because 2 individuals had missing information for their use of manufactured cigarettes.

b Data were pooled across the four iterations of the survey to increase sample size, especially for exclusive users of RYO cigarettes.

c The p-values are from chi-squared tests of independence and are testing whether the sociodemographic composition differed between the two time periods (i.e. 2010–2011 vs 2017–2018). All tests were two-tailed and deemed significant at p<0.05.

d Includes those retired, homemakers, or very disabled and unable to work, or attending school.

RYO cigarettes were associated with higher quit ratios than manufactured cigarettes within each category of race. By race, 48.9% of Indians/Asians who had ever tried RYO cigarette smoking had quit; followed by Whites (34.5%), Black Africans (21.9%), and Coloreds (15.8%). For manufactured cigarettes, quit ratios were highest among Black Africans (15.0%), followed by Whites (12.7%), Coloreds (11.0%), and Indians/Asians (7.9%).

### Multivariable analysis of factors associated with current RYO cigarette smoking during 2017–2018

Within adjusted analyses, the strongest correlates of current RYO cigarette smoking were being a current (APR=22.65; 95% CI: 11.89–43.15) or a former (APR=15.89; 95% CI: 5.97–42.31) smoker of manufactured cigarettes. Current use of hookah (APR=2.40; 95% CI: 1.57–3.68) was also significantly associated with current RYO cigarette smoking; however, no significant associations were seen between current RYO cigarette use and use of cigars, e-cigarettes, or snuff (Table 5). Those unemployed were more likely to report current use of RYO cigarettes than those employed (APR=1.70; 95% CI: 1.18–2.43). Conversely, the likelihood of reporting current RYO cigarette use was lower among females compared to males (APR=0.36; 95% CI: 0.24–0.55), urban than rural residence (APR=0.67; 95% CI: 0.46–0.97), Indians/Asians compared to Blacks (APR=0.22; 95% CI: 0.12–0.40), and among those living in Free state compared to Gauteng (APR=0.53; 95% CI: 0.30–0.96).

**Table 5:** Adjusted prevalence ratio (APR) for factors associated with current use of RYO cigarettes among South African adults aged ≥16 years, 2017–2018

*Characteristics*	*Categories*	*APR (95% CI)*	*p*
**Province**	Gauteng (Ref.)	1	
	Western Cape	0.57 (0.31–1.05)	0.07
	Eastern Cape	0.79 (0.45–1.41)	0.43
	Northern Cape	0.90 (0.46–1.74)	0.745
	Free State	0.53 (0.30–0.96)	0.035
	KwaZulu-Natal	1.14 (0.73–1.78)	0.575
	North West	0.63 (0.38–1.06)	0.08
	Mpumalanga	0.81 (0.48–1.36)	0.425
	Limpopo	0.69 (0.34–1.39)	0.295
**Age** (years)	16–24 (Ref.)	1	
	25–35	0.88 (0.59–1.33)	0.546
	35–44	0.94 (0.60–1.47)	0.783
	45–54	0.91 (0.56–1.48)	0.713
	55–64	0.79 (0.47–1.32)	0.369
	≥65	0.48 (0.21–1.11)	0.085
**Gender**	Male (Ref.)	1	
	Female	0.36 (0.24–0.55)	<0.001
**Employment status**	Employed (Ref.)	1	
	Unemployed	1.70 (1.18–2.43)	0.004
	Not in the workforce^a^	1.19 (0.77–1.83)	0.446
**Residence**	Rural (Ref.)	1	
	Urban	0.67 (0.46–0.97)	0.033
**Race**	Black (Ref.)	1	
	Colored	1.20 (0.78–1.86)	0.403
	Indian/Asian	0.22 (0.12–0.40)	<0.001
	White	0.49 (0.24–1.00)	0.051
**Smoking of manufactured cigarettes**	Non-smoker (Ref.)	1	
	Current smoker	22.65 (11.89–43.15)	<0.001
	Former smoker	15.89 (5.97–42.31)	<0.001
**Hookah smoking**	Non-smoker (Ref.)	1	
	Current smoker	2.40 (1.57–3.68)	<0.001
**Cigar smoking**	Non-smoker (Ref.)	1	
	Current smoker	1.45 (0.73–2.90)	0.288
**E-cigarette use**	Non-user (Ref.)	1	
	Current user	1.05 (0.55–2.03)	0.879
**Snuff use**	Non-user (Ref.)	1	
	Current user	1.71 (0.93–3.13)	0.082

Adjusted analysis controlled for all factors listed in table.

a Includes those retired, homemakers, or very disabled and unable to work, or attending school. RYO: roll-your-own.

### Annual expenditures associated with daily smoking of RYO versus manufactured cigarettes

Overall, within 2018 SASAS, 15.4% (n=456) were daily smokers of manufactured cigarettes. Within weighted analyses, the median cigarettes smoked per month was 210 sticks, and the median monthly expenditure was ZAR 405. Extrapolated median annual expenditures were ZAR 4860 based on daily smoking of manufactured cigarettes.

For RYO cigarettes, estimated expenditures varied by assumed amount of tobacco consumed per rolled cigarette. Based on median cigarette consumption of 2190 cigarettes per year, the estimated annual expenditures were as follows by varying paper sizes: for a 70 mm cigarette paper consuming 0.75 g of tobacco per cigarette roll, expected annual expenditure was ZAR 3194.4. For a 79 mm cigarette paper consuming 0.88 g of tobacco per roll, expected annual expenditure was ZAR 3775.2. For a 100 mm cigarette paper consuming 1.18 g of tobacco per roll, expected annual expenditure was ZAR 5420.8.

## DISCUSSION

The results of the study show that the prevalence of ever RYO cigarette use increased significantly among the South African adult population between 2010–2011 and 2017–2018, from 6.5% to 8.5%; current RYO cigarette use however remained unchanged. Current use prevalence during 2017–2018 was highest among those who self-identified as Coloreds (11.1%) and Black Africans (6.6%), aged 16–24 (7.0%) and 25–34 years (7.8%), males (11.6%), those with only primary school education (8.4%), and the unemployed (9.8%). A common link across these groups is that they are mostly price-sensitive populations who might be using RYO cigarettes as a price minimizing strategy. Indeed, our results suggest that while the annual expenditure associated with daily RYO cigarette smoking may be highly variable depending on the amount of tobacco consumed per roll, annual expenditures associated with the most commonly smoked cigarette size^20^ (i.e. King-sized cigarettes, 79–88 mm), were ZAR 3775.2, substantially less than annual expenditures for daily smoking of manufactured cigarettes, ZAR 4860. These findings are in line with previous reports from South Africa and abroad^1-6^. Nonetheless, our findings also raise the possibility that heavy RYO cigarette smoking may be associated with higher expenditures than the average smoker of manufactured cigarettes, thus limiting cash flow for household necessities among groups that are already at an economic disadvantage.

Existing disparities in the South African tobacco excise taxation system may encourage and perpetuate the use of RYO as a substitute product for cigarettes. During the 2020–2021 tax year, the excise tax on a pack of 20 cigarettes was ZAR 16.66^21^, compared to ZAR 5.39 for a 25 g bag of pipe tobacco which is also used as RYO tobacco^6^. Harmonizing tax between these products would mean raising the tax on the 25 g bag from ZAR 5.39 to 22.5 given that a 25 g pouch of tobacco contained in RYO cigarettes is approximately the same amount of tobacco as 1.35 packs of manufactured cigarettes^22^. Harmonizing tax differences on the diversity of tobacco products on the South African market may benefit public health, especially between substitute products that have similar harm profiles such as manufactured cigarettes and RYO cigarettes. This is especially important given our finding that former smokers of manufactured cigarettes had a highly elevated likelihood of reporting current use of RYO cigarettes, possibly because of reduced harm perception, easier access, and greater affordability.

In our study, exclusive RYO cigarette smokers differed from exclusive smokers of manufactured cigarettes in having a higher proportion of Black African respondents, youth, with less education, and those unemployed. Those who reported dual use of RYO and manufactured cigarettes, had some sociodemographic characteristics that fell between those of exclusive RYO and exclusive manufactured cigarettes. These might be individuals who would smoke manufactured cigarettes if they can, but RYO if they must (i.e. as a substitute product). Non-daily smoking was more prevalent for RYO than manufactured cigarettes. This may be attributable to less disposable income on the part of RYO cigarette smokers to sustain daily use, especially when considering the demographic profiles of users.

Although other studies^1,10,23^ found that quitting rates were lower among exclusive RYO cigarette smokers than exclusive smokers of manufactured cigarettes due to the low cost of RYO cigarettes and perceptions of less harm; our study results suggest that quitting rates were higher among exclusive RYO cigarette smokers than exclusive smokers of manufactured cigarettes. This may be attributed to factors such as non-daily use (possibly lower dependence), negative health effects and inconvenience associated with hand rolling^10^. There is a need for tailored smoking cessation efforts for RYO and manufactured cigarette smokers. Also, the adoption of plain packaging laws and restriction on flavors could further increase quit attempts among exclusive RYO cigarette smokers.

Some groups with relatively low prevalence during 2017–2018, saw very large increases in the prevalence of current RYO cigarette smoking between 2010–2011 and 2017–2018. For example, prevalence of current use increased from 0.5% to 4.2% among those who self-identified as White, a 669% relative percent change (RPC). Relatively large increases were also seen for those with matriculation or equivalent (4.0% to 7.1%, relative percentage change of 80%). Surveillance efforts should therefore focus, not only on groups with large prevalence, but also those with large increases over time even if their current prevalence is not dramatic. It is also important to monitor demographic shifts in the overall population over time, as those underlying shifts might influence prevalence. Knowing which groups are driving prevalence (e.g. shifts in the percentage of those unemployed) is important to develop targeted interventions to help reduce demand and use of tobacco products.

### Strengths and limitations

The strengths of this study include use of a nationally representative sample of South African adults to examine changes in prevalence of RYO cigarette smoking within the past decade. The standardized methodology of SASAS over time allows for direct comparisons of results. Nonetheless, limitations exist. First, this was a cross-sectional study; therefore, we cannot make any causal inferences. Second, a face-to-face interviewer-administered questionnaire was used to collect data, which together with self-reported risk behavior, might increase the likelihood of participants offering socially desirable responses and potentially misclassifying their smoking status. Third, there is a potential for recall bias on past smoking behavior. It is, however, unlikely that the use of self-reporting will significantly influence our measure of smoking status, as self-report has been shown to be a valid means of assessing smoking status in population surveys^24^. Fourth, annual RYO expenditures did not include costs associated with acquiring rolling machines, rolling paper, filter tips, filter tubes, lighters, holders for papers or tobacco (i.e. RYO tin), aroma cards, or other related accessories. Finally, while we attempted to capture a cross-section of RYO prices from online vendors, some vendors had removed most prices from their websites to comply with restrictions on sales of tobacco products through the postal services, the internet or any other electronic media. The captured prices may therefore not be fully representative of all RYO products sold in stores and over the internet within South Africa. Furthermore, the reported associated costs for RYO cigarettes may be overestimated as the online listed vendors may be more expensive and may not represent all sources of tobacco purchases for RYO cigarettes, especially by low socioeconomic status RYO cigarette smokers who are also more likely to use cheaper pipe tobacco than RYO tobacco.

## CONCLUSIONS

The use of RYO cigarettes was more common among Coloreds, and Black Africans. It was also common among youths, males, those with less education (primary school) and those who were unemployed. Based on typical usage, the annual expenditures associated with daily RYO cigarette smoking based on typical usage patterns were substantially less than that of smoking manufactured cigarettes. Efforts to harmonize taxation of cigarettes and RYO products may discourage use among price-sensitive populations. At the same time, intensified implementation of evidence-based tobacco prevention and control measures is needed to shift risk for the entire population as a whole and reduce aggregate tobacco consumption.

## Data Availability

The data supporting this research are available from the authors on reasonable request.
